# Supplementary material for: The burden of non-communicable disease risk factors in a low-income population: Findings from a cross-sectional study highlighting the prevalence of obesity, hypertension, and metabolic disorders in the south of Quito, Ecuador
Source: PLoS One. 2025 Sep 17;20(9):e0332159. doi: 10.1371/journal.pone.0332159 (PMC12443244; doi:10.1371/journal.pone.0332159)
Supplement: S1 File — Table S1. Definitions and cut-off points of the analysed behavioural, metabolic, and ardiovascular risk variables. Table S2. Prevalence of behavioural non-communicable disease risk factors by age. Table S3. Prevalence of metabolic non-communicable disease risk factors by age and weight status. (DOCX) [file pone.0332159.s001.docx]

| Table S1. Definitions and cut-off points of the analysed behavioural, metabolic, and ardiovascular risk variables | |  |
| --- | --- | --- |
| **Variable Name** | **Cut Points / Categories** | |
| Fruit and Vegetable Consumption | ≥5 pieces/day <5 pieces/day | |
| Physical Activity | ≥150 min/week <150 min/week | |
| Body Mass Index (BMI) | Normal: <25 kg/m²; Overweight: 25-29.9 kg/m²; Obesity: ≥30 kg/m² | |
| Waist Circumference (WC) | Men: Raised if >94 cm ; Women: Raised if >90 cm | |
| Hypertension | SBP ≥140 mmHg or DBP ≥90 mmHg or treatment for hypertension | |
| Glycaemic Status | Normoglycemia: - PGB <140 mg/dL or- FBG 71-98 mg/dL  Impaired Glucose Tolerance:PGB 140-199 mg/dL or FBG 100-125 mg/dL  Hyperglycaemia (Diabetes): - PGB >200 mg/dL or- FPG ≥126 mg/dL  Or Pharmacological treatment | |
| Total Cholesterol | Raised: >190 mg/dL  Normal: ≤190 mg/dL | |
| HDL Cholesterol | Low: - Women: ≤50 mg/dL - Men: ≤40 mg/dL | |
| LDL Cholesterol | Raised: ≥115 mg/dL Normal: <115 mg/dL | |
| Triglycerides | Raised: >150 mg/dL Normal: ≤150 mg/dL | |
| Creatinine (Normal Levels) | Men: 0.7-1.3 mg/dL Women: 0.6-1.1 mg/dL | |
| Cardiovascular (WHO CVD Risk) | Low / Moderate / High / Very High (as per WHO 2019 tables) | |
|  |  |  |

| **Age group** | **Young adults** | **Early adults** | **Middle age adults** | **Older adults** | **Total** | **p** |
| --- | --- | --- | --- | --- | --- | --- |
|  | [18-25] | (25-35] | (35-60] | >60 |  |  |
|  | N(%) | N(%) | N(%) | N(%) | N(%) | p |
| **Tobacco use** |  |  |  |  |  |  |
| Daily Smoker | 6(7.41) | 5(4.81) | 13(4.47) | 7(3.89) | 31(4.73) | 0.265 |
| Occasional Smoker | 9(11.11) | 10(9.62) | 18(6.19) | 8(4.44) | 45(6.86) |  |
| Never Smoke | 66(81.48) | 89(85.58) | 260(89.35) | 165(91.67) | 580(88.41) |  |
| **Alcohol consumption** | |  |  |  |  |  |
| Current drinker | 42(51.85) | 59(56.73) | 146(50.17) | 86(47.78) | 333(50.76) | **0.007** |
| Ex-drinker | 13(16.05) | 33(31.73) | 81(27.84) | 44(24.44) | 171(26.07) |  |
| Never Drink | 26(32.10) | 12(11.54) | 64(21.99) | 50(27.78) | 152(23.17) |  |
| **Fruit and Vegetables consumption** | | | |  |  |  |
| <5 portions per day | 67(82.72) | 86(82.69) | 263(90.38) | 164(91.11) | 580(88.41) | **0.043** |
| ≥5 portions per day | 14(17.28) | 18(17.31) | 28(9.62) | 16(8.89) | 76(11.59) |  |
| **Salt consumption** | |  |  |  |  |  |
| Low | 56(69.14) | 63(60.58) | 191(65.64) | 128(71.11) | 438(66.77) | 0.296 |
| High | 25(30.86) | 41(39.42) | 100(34.36) | 52(28.89) | 218(33.23) |  |
| **Sugar consumption** | |  |  |  |  |  |
| Low | 24(29.63) | 29(27.88) | 78(26.80) | 50(27.78) | 181(27.59) | 0.966 |
| High | 57(70.37) | 75(72.12) | 213(73.20) | 130(72.22) | 475(72.41) |  |
| **Physical activity per week** | | |  |  |  |  |
| <150 mins | 17(20.99) | 23(22.12) | 67(23.02) | 53(29.44) | 160(24.39) | 0.309 |
| ≥150 mins | 64(79.01) | 81(77.88) | 224(76.98) | 127(70.56) | 496(75.61) |  |
| **Total** | 81(100) | 104(100) | 291(100) | 180(100) | 656(100) |  |

Table S2. Prevalence of behavioural non-communicable disease risk factors by age

P Values were calculated using the Chi-square (χ²) test; Fisher’s exact test was applied when more than 20% of expected cell counts were less than 5.

Table S3. Prevalence of metabolic non-communicable disease risk factors by age and weight status

|  | **Overweight** | | | | | | **Obesity** | | | | | | **Weight status** |
| --- | --- | --- | --- | --- | --- | --- | --- | --- | --- | --- | --- | --- | --- |
| **Age group** | **Young adults** | **Early adults** | **Middle age adults** | **Older adults** | **Total** | **p** | **Young adults** | **Early adults** | **Middle age adults** | **Older adults** | **Total** | **p** | **p** |
|  | [18-25] | (25-35] | (35-60] | >60 |  |  | [18-25] | (25-35] | (35-60] | >60 |  |  |  |
| **Waist circumference** |  |  |  |  | N=234 |  |  |  |  |  | N=188 |  | N=422 |
| Normal | 9(56.25) | 25(71.43) | 59(49.58) | 19(29.69) | 112(47.86) | **0.001** | 1(10.00) | 3(12.50) | 3(3.12) | 0(0.00) | 7(3.72) | **0.035** | **<0.001** |
| Raised | 7(43.75) | 10(28.57) | 60(50.42) | 45(70.31) | 122(52.14) |  | 9(90.00) | 21(87.50) | 93(96.88) | 58(100.00) | 181(96.28) |  |  |
| **Blood pressure status** |  |  |  |  | N=234 |  |  |  |  |  | N=189 |  | N=423 |
| Normotension | 15(93.75) | 32(91.43) | 96(80.67) | 32(50.00) | 175(74.79) | **<0.001** | 10(100.00) | 23(95.83) | 67(69.07) | 22(37.93) | 122(64.55) | **<0.001** | **0.022** |
| Hypertension | 1(6.25) | 3(8.57) | 23(19.33) | 32(50.00) | 59(25.21) |  | 0(0.00) | 1(4.17) | 30(30.93) | 36(62.07) | 67(35.45) |  |  |
| **Blood glucose status** |  |  |  |  | N=233 |  |  |  |  |  | N=188 |  | N=421 |
| Normoglycemia | 16(100.00) | 30(85.71) | 87(73.73) | 34(53.12) | 167(71.67) | **0.001** | 9(90.00) | 21(91.30) | 67(69.07) | 23(39.66) | 120(63.83) | **<0.001** | 0.082 |
| Impaired tolerance | 0(0.00) | 5(14.29) | 25(21.19) | 21(32.81) | 51(21.89) |  | 0(0.00) | 2(8.70) | 21(21.65) | 22(37.93) | 45(23.94) |  |  |
| Hyperglycaemia | 0(0.00) | 0(0.00) | 6(5.08) | 9(14.06) | 15(6.44) |  | 1(10.00) | 0(0.00) | 9(9.28) | 13(22.41) | 23(12.23) |  |  |
| **Cholesterol status** |  |  |  |  | N=232 |  |  |  |  |  | N=186 |  | N=418 |
| Normal | 9(56.25) | 23(65.71) | 42(35.90) | 14(21.88) | 88(37.93) | **<0.001** | 33(60.00) | 23(62.50) | \|(34.74) | 77(40.35) | 77(41.40) | 0.055 | 0.471 |
| Hypercholesterolemia | 7(43.75) | 12(34.29) | 75(64.10) | 50(78.12) | 144(62.07) |  | 4(40.00) | 9(37.50) | 62(65.26) | 34(59.65) | 109(58.60) |  |  |
| **HDL** |  |  |  |  | N=232 |  |  |  |  |  | N=186 |  | N=418 |
| Lower | 2(12.50) | 5(14.29) | 41(35.04) | 35(54.69) | 83(35.78) | **<0.001** | 1(10.00) | 5(20.83) | 29(30.53) | 25(43.86) | 60(32.26) | 0.062 | 0.451 |
| Normal | 14(87.50) | 30(85.71) | 76(64.96) | 29(45.31) | 149(64.22) |  | 9(90.00) | 19(79.17) | 66(69.47) | 32(56.14) | 126(67.74) |  |  |
| **LDL** |  |  |  |  | N=223 |  |  |  |  |  | N=178 |  | N=401 |
| Normal | 8(50.00) | 26(74.29) | 49(44.55) | 21(33.87) | 104(46.64) | **0.002** | 7(77.78) | 16(69.57) | 35(38.46) | 27(49.09) | 85(47.75) | **0.013** | 0.824 |
| Raised | 8(50.00) | 9(25.71) | 61(55.45) | 41(66.13) | 119(53.36) |  | 2(22.22) | 7(30.43) | 56(61.54) | 28(50.91) | 93(52.25) |  |  |
| **Triglycerides** |  |  |  |  | N=232 |  |  |  |  |  | N=186 |  | N=418 |
| Normal | 6(37.50) | 17(48.57) | 42(35.90) | 30(46.88) | 95(40.95) | 0.381 | 5(50.00) | 12(50.00) | 34(35.79) | 22(38.60) | 73(39.25) | 0.530 | 0.724 |
| Raised | 10(62.50) | 18(51.43) | 75(64.10) | 34(53.12) | 137(59.05) |  | 5(50.00) | 12(50.00) | 61(64.21) | 35(61.40) | 113(60.75) |  |  |
| **Seric Creatinine** |  |  |  |  | N=232 |  |  |  |  |  | N=186 |  | N=418 |
| Lower | 15(93.75) | 31(88.57) | 101(86.32) | 50(78.12) | 197(84.91) | **0.022** | 8(80.00) | 20(83.33) | 80(84.21) | 47(82.46) | 155(83.33) | 0.173 | 0.376 |
| Normal | 0(0.00) | 0(0.00) | 1(0.85) | 8(12.50) | 9(3.88) |  | 0(0.00) | 0(0.00) | 0(0.00) | 4(7.02) | 4(2.15) |  |  |
| Raised | 1(6.25) | 4(11.43) | 15(12.82) | 6(9.38) | 26(11.21) |  | 2(20.00) | 4(16.67) | 15(15.79) | 6(10.53) | 27(14.52) |  |  |
| **Total** | 16(100) | 35(100) | 119(100) | 64(100) |  |  | 10(100) | 24(100) | 97(100) | 58(100) |  |  |  |
| P Values were calculated using the Chi-square (χ²) test; Fisher’s exact test was applied when more than 20% of expected cell counts were less than 5. | | | | | | | | | | | | | |
