# Supplementary material for: The burden of non-communicable disease risk factors in a low-income population: Findings from a cross-sectional study highlighting the prevalence of obesity, hypertension, and metabolic disorders in the south of Quito, Ecuador
Source: PLoS One. 2025 Sep 17;20(9):e0332159. doi: 10.1371/journal.pone.0332159 (PMC12443244; doi:10.1371/journal.pone.0332159)
Supplement: S2 File — (PDF) [file pone.0332159.s002.pdf]

INTRODUCCIÓN ENCUESTA

❖ Identificación del entrevistador

❖ Nombre del punto de geolocalización

❖ Fecha en que se cumplimentó el cuestionario

(yyyy-mm-dd)

❖ Personas mayores de 18 años viviendo en el domicilio (reside al menos 20 días al mes y no tiene planes de mudanza en el futuro cercano)

Antes de pasar a la siguiente pregunta ANOTAR EN UNA HOJA DE EXCEL el nombre de pila de las personas mayores de 18 años y asignar a cada nombre un número consecutivo: 1, 2, 3...

La persona seleccionada es la número ''

❖ ¿Se encuentra la persona seleccionada en casa?

◯**Sí**
◯**No**

❖ ¿Podría disponer de un número de teléfono personal para contactar a la persona?

Si no es posible obtener el número móvil, preguntar cuándo sería posible encontrar a la persona.

Escanear QR del consentimiento

❖ Se leyó el texto del consentimiento al entrevistado y este dio su consentimiento

Si la respuesta es «No», entregar consentimiento de negación y terminar encuesta.

◯**Sí**
◯**No**

❖ Idioma de la entrevista

◯**Español**
◯**Otro**

❖ Si la respuesta anterior es «Otro», ¿cuál?

❖ Hora de la entrevista

(sistema 24 horas)
hh:mm

❖ Apellido

❖ Nombre de pila

❖ Número de teléfono para contacto, siempre que sea posible

- Sexo

(anote masculino o femenino SEGÚN LO QUE OBSERVE)

◯Masculino
◯Femenino

- ¿En qué fecha nació usted?

(dd/mm/aaaa) / SI NO SABE: 77/77/7777

- ¿Qué edad tiene usted?

(en años) SI NO SABE: 777

- ¿Cuántos años en total asistió a la escuela a tiempo completo (sin contar la etapa preescolar)?

SI NO SABE: 777

- ¿Cuál es el grado más alto de escolaridad que alcanzó usted?

◯No tuvo instrucción formal
◯No terminó la primaria
◯Terminó la primaria
◯Terminó la secundaria
◯Terminó la etapa preuniversitaria
◯Terminó la universidad o enseñanza superior
◯Tiene un posgrado
◯Se negó a responder

- ¿Cómo se identifica usted según su cultura y costumbres?

(grupo étnico o racial, subgrupo cultural u otra colectividad semejante)

◯Indígena
◯Afrodescendiente
◯Negro
◯Mulato
◯Montubio
◯Mestizo
◯Blanco
◯Otro
◯Se negó a responder

- ¿Cuál es su estado civil?

◯Nunca se ha casado
◯Actualmente casado o casada
◯Separado o separada
◯Divorciado o divorciada
◯Viudo o viuda

◯Unión libre
◯Se negó a responder

 ¿Cuál de las frases siguientes describe mejor su situación laboral en los últimos 12 meses?

Muestre la ayuda gráfica

◯Funcionario público
◯Empleado
◯Trabaja por cuenta propia
◯Trabaja sin remuneración
◯Estudia
◯Ama de casa
◯Jubilado o jubilada
◯Desempleado o desempleada (en condiciones de trabajar)
◯Desempleado o desempleada (incapaz de trabajar)
◯Se negó a responder

 Además de usted ¿cuántas personas mayores de 18 años viven en su hogar?

 Teniendo en cuenta el año pasado, ¿puede usted decirme cuáles fueron los ingresos medios mensuales de la familia?

SI NO SABE: 7 / SI SE NIEGA A RESPONDER: 9

 Si no conoce la cantidad exacta, ¿podría darnos una estimación del ingreso familiar anual si leo en voz alta algunas opciones? La cantidad es de:

FALTA: INTRODUCIR QUINTILES EN MONEDA LOCAL (Leer las opciones)

◯≤Quintil (Q) 1
◯¿Más que el Q1 pero ≤Q2
◯¿Más que el Q2 pero ≤Q3
◯¿Más que el Q3 pero ≤Q4
◯¿Más que el Q4?
◯No sabe
◯Se negó a responder

CONSUMO DE TABACO

Quisiera hacerle algunas preguntas sobre el consumo de tabaco. Muestre la ayuda gráfica

 ¿Fuma usted actualmente algún producto de tabaco como cigarrillos, puros o pipa?

Muestre la ayuda gráfica

◯Sí
◯No

 ¿Fuma usted actualmente productos de tabaco todos los días?

◯Sí
◯No

 ¿Qué edad tenía usted cuando empezó a fumar?

SI NO SABE: 777

 ¿Recuerda usted hace cuántos AÑOS fue?

 ¿Recuerda usted hace cuántos MESES fue?

 ¿Recuerda usted hace cuántas SEMANAS fue?

 Ahora le voy a preguntar por su frecuencia de consumo de distintos productos.

- ¿Con qué frecuencia fuma usted cigarrillos fabricados?

Muestre la ayuda gráfica

◯A diario
◯Semanalmente
◯Nunca

- ¿Cuántos cigarrillos fabricados fuma?

HAGA REFERENCIA A LA FRECUENCIA ESPECIFICADA EN LA PREGUNTA ANTERIOR (a diario / semanalmente)

- ¿Con qué frecuencia fuma usted cigarrillos liados a mano?

Muestre la ayuda gráfica

◯A diario
◯Semanalmente
◯Nunca

- ¿Cuántos cigarrillos liados a mano fuma?

HAGA REFERENCIA A LA FRECUENCIA ESPECIFICADA EN LA PREGUNTA ANTERIOR (a diario / semanalmente)

- ¿Con qué frecuencia fuma usted pipas llenas?

Muestre la ayuda gráfica

◯A diario
◯Semanalmente
◯Nunca

- ¿Cuántas pipas llenas fuma?

HAGA REFERENCIA A LA FRECUENCIA ESPECIFICADA EN LA PREGUNTA ANTERIOR (a diario / semanalmente)

- ¿Con qué frecuencia fuma usted puros o puritos?

Muestre la ayuda gráfica

◯A diario
◯Semanalmente
◯Nunca

- ¿Cuántos puros o puritos fuma?

HAGA REFERENCIA A LA FRECUENCIA ESPECIFICADA EN LA PREGUNTA ANTERIOR (a diario / semanalmente)

- ¿Con qué frecuencia tiene usted sesiones de pipa de agua?

Muestre la ayuda gráfica

◯A diario
◯Semanalmente
◯Nunca

- ¿Cuántas sesiones de pipa de agua?

HAGA REFERENCIA A LA FRECUENCIA ESPECIFICADA EN LA PREGUNTA ANTERIOR (a diario / semanalmente)

- ¿Fuma usted otro tipo de tabaco?

◯Sí
◯No

- ¿Con qué frecuencia fuma usted este otro tipo de tabaco?

◯A diario
◯Semanalmente
◯Nunca

- ¿Puede especificar qué tipo de tabaco?

- ¿Cuánto fuma de este tipo de tabaco?

 En los últimos 12 meses, ¿ha tratado de dejar de fumar?

◯Sí
◯No

 En los últimos 12 meses, ¿le han aconsejado que deje de fumar en alguna visita al médico u otro agente sanitario?

◯Sí
◯No
◯No ha visitado a ningún médico o agente sanitario en los últimos 12 meses

 ¿Fumó usted anteriormente?

◯Sí
◯No

 ¿Anteriormente llegó usted a fumar todos los días?

◯Sí
◯No

CONSUMO DE ALCOHOL

 ¿Alguna vez ha consumido bebidas alcohólicas, como cerveza, vino, licor, chicha o miske?
Muestre la ayuda gráfica o dé ejemplos
◯Sí
◯No

 En los últimos 12 meses, ¿ha consumido alcohol?
◯Sí
◯No

 ¿Ha dejado de beber por motivos de salud, porque perjudica su salud o por consejo del médico u otro agente sanitario?
◯Sí
◯No

▪ En los últimos 12 meses, ¿con que frecuencia ha consumido por lo menos una bebida alcohólica corriente?
◯Todos los días
◯Entre 5 y 6 días por semana
◯Entre 3 y 4 días por semana
◯Entre 1 y 2 días por semana
◯Entre 1 y 3 días por mes
◯Menos de una vez al mes

▪ En los últimos 30 días, ¿ha consumido cualquier tipo de alcohol?
◯Sí
◯No

▪ En los últimos 30 días, ¿en cuántas ocasiones consumió usted por lo menos un trago de una bebida alcohólica corriente?
SI NO SABE: 777

▪ En los últimos 30 días, cuando bebió alcohol, ¿cuántos tragos ordinarios, en promedio, consumió en cada ocasión?
Muestre la ayuda gráfica, SI NO SABE: 777

▪ En los últimos 30 días, ¿cuál fue el mayor número de tragos que bebió en una sola ocasión, sumando todos los tipos de bebidas alcohólicas?
SI NO SABE: 777

- En los 30 últimos días, ¿cuántas veces ha bebido seis o más tragos ordinarios en una sola ocasión?
  SI NO SABE: 777

- En la última semana, ¿cuántos tragos ordinarios bebió usted el LUNES?
  SI NO SABE: 777

- En la última semana, ¿cuántos tragos ordinarios bebió usted el MARTES?
  SI NO SABE: 777

- En la última semana, ¿cuántos tragos ordinarios bebió usted el MIÉRCOLES?
  SI NO SABE: 777

- En la última semana, ¿cuántos tragos ordinarios bebió usted el JUEVES?
  SI NO SABE: 777

- En la última semana, ¿cuántos tragos ordinarios bebió usted el VIERNES?
  SI NO SABE: 777

- En la última semana, ¿cuántos tragos ordinarios bebió usted el SÁBADO?
  SI NO SABE: 777

- En la última semana, ¿cuántos tragos ordinarios bebió usted el DOMINGO?
  SI NO SABE: 777

Le acabo de preguntar por su consumo de alcohol en los últimos 7 días. Me refería a las bebidas alcohólicas en general; las preguntas siguientes serán acerca del consumo de bebidas alcohólicas elaboradas en casa, del alcohol que viene del otro lado de la frontera o de otro país, y de cualquier forma de alcohol que no es para beber o no paga impuestos. Cuando responda a las preguntas que siguen, concéntrese únicamente en los tipos de alcohol que acabo de mencionar.

- En los últimos 7 días, ¿consumió usted alguna bebida alcohólica elaborada en casa, alguna bebida alcohólica procedente del otro lado de la frontera o de otro país, algún tipo de alcohol que no es apto para beberse u otra forma de alcohol que no paga impuestos?
  - ◯ Sí
  - ◯ No

- En promedio, ¿cuántos tragos ordinarios de LICORES DE ELABORACIÓN DOMESTICA bebió usted en los últimos 7 días?
  (Chicha, guarapo…)

- En promedio, ¿cuántos tragos ordinarios de cerveza o vino de elaboración casera, incluido el vino de palma o el de frutas bebió usted en los últimos 7 días?
  (Muestre la ayuda gráfica) SI NO SABE: 77

- En promedio, ¿cuántos tragos ordinarios de bebida alcohólica traída del otro lado de la frontera o de otro país bebió usted en los últimos 7 días?
  (Muestre la ayuda gráfica) SI NO SABE: 77

- En promedio, ¿cuántos tragos ordinarios de alcohol que no está destinado al consumo, como los medicamentos a base de alcohol, perfumes, lociones para después de afeitar bebió usted en los últimos 7 días?
  (Muestre la ayuda gráfica) SI NO SABE: 77

- En promedio, ¿cuántos tragos ordinarios de otro producto de alcohol que no paga impuestos en el país bebió usted en los últimos 7 días?
  (Muestre la ayuda gráfica) SI NO SABE: 77

ALIMENTACIÓN

Utilizar ayuda gráfica para mostrar ejemplos de frutas y verduras y tamaño de porción

- En una semana corriente, ¿cuántos días come usted frutas?
  (MUESTRE LA AYUDA GRÁFICA)
  - Número de días

- ¿Cuántas porciones de fruta come en uno de esos días?
  (MUESTRE LA AYUDA GRÁFICA)
  - Número de porciones

- En una semana corriente, ¿cuántos días come usted verduras y hortalizas?
  (MUESTRE LA AYUDA GRÁFICA)
  - Número de días

- ¿Cuántas porciones de verduras come usted en uno de esos días?
  (MUESTRE LA AYUDA GRÁFICA)
  - Número de porciones

- ¿Por medio, cuantas comidas por semana come algo que no fue preparado en casa? Comidas implica desayuno, almuerzo y cena.
  - Número

***SECCIÓN AÑADIDA** (En base a datos del ENSANUT, Cuestionario de frecuencia de consumo de alimentos validado en población adulta en Ecuador y clasificación de alimentos consumidos en Ecuador y estudio realizado sobre factores de riesgo dietético para enfermedades no transmisibles en Kenia utilizando la encuesta STEPS)

- ¿Suele usted agregar sal a la comida antes de comer o mientras come?
  - ◯ Siempre
  - ◯ Algunas veces
  - ◯ Nunca/ raramente
  - ◯ No sabe

- En una semana corriente, ¿cuántos días consume usted alimentos procesados con alto contenido de sal? Por ejemplo: bocadillos salados envasados, alimentos salados enlatados que incluyen encurtidos y conservas, alimentos salados preparados en un restaurante de comida rápida, queso, tocino y carne procesada. Snacks salados, snacks empaquetados, platos congelados preelaborados.
  - Número de días

- ¿Suele usted agregar azúcar a sus bebidas ya servidas?
  - ◯ Siempre
  - ◯ Algunas veces
  - ◯ Nunca/ raramente
  - ◯ No sabe

- En una semana corriente, ¿Cuantos días toma usted gaseosas o bebidas azucaradas?
  Por ejemplo: gaseosas (incluyendo las light), energizantes, jugos con azúcar.
  - Número de días

- En una semana corriente, ¿Cuantos días consume usted dulces?
  Por ejemplo: galletas, pasteles, dulces, caramelos, mermeladas, helados, chocolate y similares.
  - Número de días.

ACTIVIDAD FÍSICA

- ¿Exige su trabajo una actividad física intensa que implica una aceleración importante de la respiración o del ritmo cardíaco, como [levantar pesos, cavar o trabajos de construcción] durante al menos 10 minutos consecutivos? (INSERTAR EJEMPLOS Y UTILIZAR LAS CARTILLAS DE IMÁGENES)
  - ◯Sí
  - ◯No

- En una semana típica, ¿cuántos días realiza usted actividades físicas intensas en su trabajo?

- En uno de esos días en los que realiza actividades físicas intensas, ¿cuánto tiempo suele dedicar a esas actividades?
  (Especificar respuesta en horas y minutos HH:MM)

- ¿Exige su trabajo una actividad de intensidad moderada que implica una ligera aceleración de la respiración o del ritmo cardíaco, como caminar deprisa [o transportar pesos ligeros] durante al menos 10 minutos consecutivos? (INSERTAR EJEMPLOS Y UTILIZAR LAS CARTILLAS DE IMÁGENES)
  - ◯Sí
  - ◯No

- En una semana típica, ¿cuántos días realiza usted actividades de intensidad moderada en su trabajo? ____

- En uno de esos días en los que realiza actividades físicas de intensidad moderada, ¿cuánto tiempo suele dedicar a esas actividades?
  (Especificar respuesta en horas y minutos HH:MM)

- ¿Camina usted o usa usted una bicicleta al menos 10 minutos consecutivos en sus desplazamientos?
  - ◯Sí
  - ◯No

- En una semana típica, ¿cuántos días camina o va en bicicleta al menos 10 minutos consecutivos en sus desplazamientos?

- En un día típico, ¿cuánto tiempo pasa caminando o yendo en bicicleta para desplazarse?
  (Especificar respuesta en horas y minutos HH:MM)

- ¿En su tiempo libre, practica usted deportes/fitness intensos que implican una aceleración importante de la respiración o del ritmo cardíaco como [correr, jugar al fútbol]] durante al menos 10 minutos consecutivos? (INSERTAR EJEMPLOS Y UTILIZAR LAS CARTILLAS DE IMÁGENES)
  - ◯Sí
  - ◯No
- En una semana típica, ¿cuántos días practica usted deportes/fitness intensos en su tiempo libre?
- En uno de esos días en los que practica deportes/fitness intensos, ¿cuánto tiempo suele dedicar a esas actividades?
  (Especificar respuesta en horas y minutos HH:MM)
- ¿En su tiempo libre practica usted alguna actividad de intensidad moderada que implica una ligera aceleración de la respiración o del ritmo cardíaco, como caminar deprisa, [ir en bicicleta, nadar, jugar al voleibol] durante al menos 10 minutos consecutivos? (INSERTAR EJEMPLOS Y UTILIZAR LAS CARTILLAS DE IMÁGENES)
  - ◯Sí
  - ◯No
- En una semana típica, ¿cuántos días practica usted actividades físicas de intensidad moderada en su tiempo libre?
- En uno de esos días en los que practica actividades físicas de intensidad moderada, ¿cuánto tiempo suele dedicar a esas actividades?
  (Especificar respuesta en horas y minutos HH:MM)
- ¿Cuánto tiempo suele pasar sentado o recostado (sin incluir el tiempo de sueño) en un día típico?
  (Especificar respuesta en horas y minutos HH:MM)

OTROS: TENSIÓN ARTERIAL, DIABETES, COLESTEROL:

- ¿Alguna vez le ha medido la presión arterial un médico u otro agente sanitario?
  ◯Sí
  ◯No

- ¿Alguna vez le ha dicho un médico u otro agente sanitario que tiene usted la presión arterial alta o hipertensión arterial?
  ◯Sí
  ◯No

- ¿Se lo han dicho en los últimos 12 meses?
  ◯Sí
  ◯No

- En las dos últimas semanas, ¿ha tomado usted algún medicamento (medicina) para tratar la hipertensión arterial, que haya sido recetado por un médico u otro agente sanitario?
  ◯Sí
  ◯No

- ¿Alguna vez ha consultado usted a un curandero tradicional por la presión arterial alta o hipertensión?
  ◯Sí
  ◯No

- ¿Toma usted actualmente algún remedio herbario o tradicional contra la presión arterial alta?
  ◯Sí
  ◯No

- ¿Alguna vez le ha medido el azúcar de la sangre un médico u otro agente sanitario?
  ◯Sí
  ◯No

- ¿Alguna vez le ha dicho un médico u otro agente sanitario que tiene usted elevada el azúcar de la sangre o diabetes?
  ◯Sí
  ◯No

- ¿Se lo han dicho en los últimos 12 meses?

◯Sí
◯No

- En las dos últimas semanas, ¿ha tomado usted algún medicamento (remedio) para tratar la diabetes, que haya sido recetado por un médico u otro agente sanitario?
  ◯Sí
  ◯No

- ¿Actualmente recibe usted insulina contra la diabetes, recetada por un médico u otro agente sanitario?
  ◯Sí
  ◯No

- ¿Alguna vez ha consultado usted a un curandero tradicional por la diabetes o azúcar de la sangre elevada?
  ◯Sí
  ◯No

- ¿Toma usted actualmente algún remedio herbario o tradicional contra la diabetes?
  ◯Sí
  ◯No

- ¿Alguna vez le ha medido el colesterol (un tipo de grasa en la sangre) un médico u otro agente sanitario?
  ◯Sí
  ◯No

- ¿Alguna vez le ha dicho un médico u otro agente sanitario que tiene usted elevado el colesterol sanguíneo?
  ◯Sí
  ◯No

- ¿Se lo han dicho en los últimos 12 meses?
  ◯Sí
  ◯No

- En las dos últimas semanas, ¿ha tomado usted algún medicamento (remedio) oral para tratar el colesterol elevado, que haya sido recetado por un médico u otro agente sanitario?
  ◯Sí

◯No

- ¿Alguna vez ha consultado usted a un curandero tradicional por el colesterol elevado?

  ◯Sí
  ◯No

- ¿Toma usted actualmente algún remedio herbario o tradicional contra el colesterol elevado?

  ◯Sí
  ◯No

- ¿Alguna vez ha sufrido usted un ataque cardiaco o dolor de pecho causado por una enfermedad del corazón (angina de pecho) o un ataque cerebral (accidente cerebrovascular, apoplejía)?

  ◯Sí
  ◯No

- ¿Actualmente toma usted regularmente ácido acetilsalicílico (aspirina) para prevenir o tratar una enfermedad del corazón?

  ◯Sí
  ◯No

- ¿Actualmente toma usted regularmente alguna «estatina» (lovastatina, sinvastatina, atorvastatina u otra) para prevenir o tratar una enfermedad del corazón?

  ◯Sí
  ◯No

- En los últimos tres años, ¿algún médico u otro agente sanitario le ha aconsejado dejar de fumar o no empezar a fumar?

  ◯Sí
  ◯No

- En los últimos tres años, ¿algún médico u otro agente sanitario le ha aconsejado reducir el consumo de sal?

  ◯Sí
  ◯No

- En los últimos tres años, ¿algún médico u otro agente sanitario le ha aconsejado comer por lo menos cinco raciones de frutas o verduras todos los días?

  ◯Sí
  ◯No

- En los últimos tres años, ¿algún médico u otro agente sanitario le ha aconsejado reducir el consumo de grasa?
  - ◯Sí
  - ◯No

- En los últimos tres años, ¿algún médico u otro agente sanitario le ha aconsejado comenzar a realizar actividad física o aumentarla?
  - ◯Sí
  - ◯No

- En los últimos tres años, ¿algún médico u otro agente sanitario le ha aconsejado mantener un peso sano o adelgazar?
  - ◯Sí
  - ◯No
  - ◯No sé

- ¿Está usted embarazada o cree que podría estarlo?

  Si tiene dudas, le ofrecemos la posibilidad de realizar un test de embarazo gratuitamente y que nos diga el resultado el día de la prueba y toma de medidas.
  - ◯Sí
  - ◯No
  - ◯No sé

- ¿Puede facilitarnos un teléfono de contacto para concertar la cita para la prueba y toma de medidas?
