# Supplementary material for: The burden of non-communicable disease risk factors in a low-income population: Findings from a cross-sectional study highlighting the prevalence of obesity, hypertension, and metabolic disorders in the south of Quito, Ecuador
Source: PLoS One. 2025 Sep 17;20(9):e0332159. doi: 10.1371/journal.pone.0332159 (PMC12443244; doi:10.1371/journal.pone.0332159)
Supplement: Attachment — Submitted filename: REVISOR_ARTICULO_MCH.docx [file pone.0332159.s004.docx]

The authors present a study on the prevalence of CNCD risk factors in a low-income population. While the STEP methodology, applied nationwide in Ecuador (2018), already incorporated stratification by area of residence (urban/rural), which presumably encompassed low-resource populations, the manuscript should more clearly justify the rationale for this specific study. What particular aspects of the investigated low-income population were not adequately addressed by the national STEPS study? Questions and suggestions for the manuscript are presented below.

Although the findings from the sample (n=656) are consistent with the data from the national sample (n=4,638), their capacity to contribute novel information appears limited. It is suggested that the analysis of sociodemographic characteristics be reoriented towards greater contextual relevance. Instead of the sex-based distribution presented in Table 1, considering the distribution by age and educational attainment could reveal significant inequalities. Furthermore, analyzing the interplay between educational attainment, employment status (employed/unemployed), and gender would facilitate a more comprehensive understanding of labor inclusion challenges within this setting. Isolated data, such as the percentage of female employment in the public sector, without stratification by educational level, lacks the necessary depth to inform context-sensitive intervention strategies for low-resource settings.

This is even more so when their conclusions state the following: "The established links between hypercholesterolaemia, diabetes, and hypertension with unhealthy patterns and pathogenic environments suggest that actions should be defined to address the social determinants of health, incorporating insights from various fields to reduce inequalities, particularly gender inequalities, and create fair environments that promote and improve health of men and women"

It would be valuable to consider age stratification with ranges allowing for enhanced granularity in the prevalence analysis, particularly within groups exhibiting dynamic consumption patterns (Table 2). For instance, disaggregating age cohorts into Young Adults (18-25 y), Early Adults (25-35 y), Middle-Aged Adults (35-60 y), and Older Adults (60 years and older) would be beneficial. Specifically, further disaggregation of the 18-40 year range into narrower subgroups, such as 18-25 years, could elucidate specific trends in young adulthood, a stage where, as noted, consumption is often influenced by autonomy, university/work life, and social determinants, including risky behaviors like binge drinking and social smoking.

To enhance the interpretation of metabolic risk factor outcomes (Table 3), additional stratification by nutritional status, beyond age cohorts, would be valuable. As evidenced by prior research, including recent studies in the Ecuadorian population lipid profiles and other metabolic parameters can vary significantly according to nutritional status. Incorporating this stratification could facilitate the identification of specific subpopulations within age ranges exhibiting distinct metabolic risk profiles, thereby informing more targeted interventions.

Ref: Albuja Quintana, Natalia (2025) Relationship between plasma uric acid levels, antioxidant capacity, and oxidative damage markers in overweight and obese adults: A cross-sectional study. <https://doi.org/10.1371/journal.pone.0312217>

In the discussion section, the authors excessively elaborate on mechanisms that their own data cannot substantiate, as exemplified in lines 295-305, 312-318, 328-336, and 341-345. Given the cross-sectional design and the limited scope of statistical analyses, mechanistic explanations are challenging to support without additional experimental evidence. It would be more appropriate for the authors to reorganize the discussion section based on their own findings to clearly articulate the novelties or actual conclusions of this study to the readers.

Starting on line 73, the bibliographic citation has a different format that should be corrected, from ref 12 to ref 17.

Line 341: The information is repeated

These findings highlight. These findings highlight the high burden o…

Figure 2: Does it not describe the units of measurement for each parameter, for example, total cholesterol is in mg/dl?
