# Supplementary material for: The burden of non-communicable disease risk factors in a low-income population: Findings from a cross-sectional study highlighting the prevalence of obesity, hypertension, and metabolic disorders in the south of Quito, Ecuador
Source: PLoS One. 2025 Sep 17;20(9):e0332159. doi: 10.1371/journal.pone.0332159 (PMC12443244; doi:10.1371/journal.pone.0332159)
Supplement: Attachment — Submitted filename: Response_to_reviewers.docx [file pone.0332159.s005.docx]

**We would like to sincerely thank both reviewers for their time and careful reading of our manuscript and for providing insightful and constructive comments. Your observations and suggestions are valuable and have helped us to improve the quality and clarity of our study.**

Reviewer 1*: “The authors are grateful for presenting a research paper in a Latin American country. The introduction considers the international contextual aspects as appropriate, emphasizing the lack of data in LAC. The aim was to describe the prevalence of risk factors for chronic diseases in a low-income district in southern Quito. It is not clear why this area is being studied.”*

*“A better characterization of the study site in terms of environmental, meteorological, and social aspects would be helpful; a map would be helpful.”*

**This study is part of the European CEAD project, which aims to provide rigorous epidemiologic data on diabetes risk and morbidity in both urban and rural low-income districts of Ecuador. We included a broader description of the CEAD project's objectives in lines 92–94 and the rationale for selecting Quito as the study setting in lines 110–118. We also included a figure (Figure 1) which includes a map of described locations.**

It is recommended that Figure 1 detail the characteristics of the GPS points that were eliminated and the participants who refused to participate.

**We broadened our description of eliminated points in the figure. We also included in the Methods (lines 142-147) and in discussion (Lines 398-400), that the recruitment was done during the pandemic, which affected how people participated.**

The Recruitment and Procedures section specifies that the cultural adaptations made to the questionnaires be detailed.

**We included a paragraph in lines 179-183 describing the cultural adaptations. We did not make major changes to the original questionnaire. Instead, we adapted the concepts and the nature of the questions to fit the Ecuador context, as per the STEP guidelines (*World Health Organization, 2006*). For example, in the sociodemographic section, the educational level scale differs slightly, we also adapted job types considering the Ecuadorian context and we included some visuals items (photos) related to the most common types of fruits and vegetables.**

- ***World Health Organization. Noncommunicable Diseases and Mental Health Cluster. Manual de vigilancia STEPS de la OMS: el método STEPwise de la OMS para la vigilancia de los factores de riesgo de las enfermedades crónicas [Internet]. World Health Organization; 2006 [cited 2023 Feb 7]. Report No.: WHO/NMH/CHP/SIP/05.02. Available from: https://apps.who.int/iris/handle/10665/43580***

In line 157, the criteria for eligible participants for the glucose tolerance test are not clear.

**We have improved the explanation in lines 189–202 regarding the process for selecting participants prior to the laboratory tests.**

References for the section are missing between lines 160 and 164

**We have included the reference “Zhou J, Fabros A, Lam SJ, Coro A, Selvaratnam R, Brinc D, Di Meo A. The stability of 65 biochemistry analytes in plasma, serum, and whole blood. Clin Chem Lab Med. 2024 Mar 7;62(8):1557-1569. doi: 10.1515/cclm-2023-1192. PMID: 38443327.” in lines 197 that details the management and treatment of the biological samples.**

It is essential to complete the data analysis section. It is not clear why multivariate analyses are not performed to identify risk factors associated with the higher prevalence rates determined. In Tables 1, 2, and 3, it is suggested to add whether the differences according to sex are significant or not, detailing the hypothesis tests used.

**We have revised the data analysis section to reflect improvements. We fully agree that the initial analysis was relatively simple aiming to describe the general health condition of participants.**

**We did not conduct multivariable analyses for each of the risk factors due to the large number of variables (both outcome variables and potential explanatory variables) and the volume of data involved. For examples, we could develop a separate multivariable model for each risk factor (behavioural and metabolic), using the population’s sociodemographic characteristics as potential explanatory variables, but we do not believe it is feasible to incorporate all these analyses within a single paper.**

**We do agree, however, that this type of analysis is important, and in fact, different members of our international team are currently undertaking such analyses, each focusing on specific research questions. For this study, our primary aim was to describe the prevalence of numerous NCD risk factors in this low-income urban setting, and as such we prioritized a descriptive approach over multivariate analyses. We acknowledge this as a limitation and have clarified it in the discussion.**

**Nevertheless, we agree that it is important to include p-values in Tables 1, 2, and 3 to indicate the statistical significance of differences observed between variables. We now present the p values for age-group (stratified by sex) and the comparison according to sex, as suggested by the reviewer. The hypothesis tests used are now detailed in the methods section.**

In the discussion section, other aspects that could be related to the determined prevalence rates are not addressed. What is the importance of the environmental characteristics that may be related to the chronic diseases indicated? For example, what is the role of altitude and air pollution?

**We have included in the discussion section (lines 364–365, 381-383) references that highlight how environmental characteristics—such as low levels of urbanisation, low pollution, and access to green spaces, which are typical of rural high-altitude communities—may protect against chronic diseases. In contrast, in the south of Quito, increasing urbanisation, higher air pollution, and other environmental factors may be contributing to the higher prevalence rates observed.**

The conclusions do not state possible interventions or public health actions that would allow the prevalence rates obtained to be reduced.

**We have included possible public health interventions in the conclusion (lines 407–423), highlighting the need to address structural barriers like poverty, limited access to specialist health professionals, and unhealthy food availability, while also considering gendered care roles and social inequalities affecting self-care.**

Reviewer #2: The authors present a study on the prevalence of CNCD risk factors in a low-income population. While the STEP methodology, applied nationwide in Ecuador (2018), already incorporated stratification by area of residence (urban/rural), which presumably encompassed low-resource populations, the manuscript should more clearly justify the rationale for this specific study. What aspects of the investigated low-income population were not adequately addressed by the national STEPS study?

**Thank you for your observation. As we noted in our response to Reviewer 1, our manuscript is part of a broader European-funded project that seeks to describe the burden of risk factors for NCDs with a particular focus on type 2 diabetes mellitus in specific low-income contexts. While we acknowledge that the national STEPS survey conducted in Ecuador in 2018 incorporated urban/rural stratification, it does not provide a picture of urban poor populations. Our study aims to complement national-level data by offering a contextualized analysis that focuses on a specific low-income urban population and offers a detailed presentation of their socioeconomic characteristics. This localized perspective is essential for understanding how NCD risk factors manifest in economically vulnerable contexts. Evaluating local realities can be useful for translating public health recommendations into context-specific actions where standard national data may overlook key local aspects.**

**At a broader level, our findings may be relevant for researchers, health professionals, and policymakers working in other contexts, as they reveal the frequent presence and coexistence of multiple risk factors within a population characterised by low socioeconomic conditions. This challenges the effectiveness of vertical programs that address individual risk factors in isolation and highlights the potential value of adopting a more ecological and integrated approach, which could lead to greater public health impact. We have broadened our description of the rationale of the study within the context of the CEAD project and provided further details on the study district.**

Questions and suggestions for the manuscript are presented below.

- Although the findings from the sample (n=656) are consistent with the data from the national sample (n=4,638), their capacity to contribute novel information appears limited. It is suggested that the analysis of sociodemographic characteristics be reoriented towards greater contextual relevance.

**Although our findings are consistent with those of the national survey, we emphasise several alarming patterns that add contextual relevance by highlighting the frequency of NCD risk factors in an urban underserved population.**

**After conducting univariate and multivariate regression models for each risk factor (behavioural and metabolic), we chose to present a descriptive analysis based on sex and age. This decision was based on our observation that, except in the case of obesity, variables such as education level introduced misleading effects on the results (older adults have more NCD risk factors but they are also the individuals with low level of education, please see the table included at the end of this response). To properly consider the socioeconomic characteristics of the population and how they influence the NCD risk factors, we would need to include a multivariable analysis to consider potential confounding. As mentioned above, in relation to comments from reviewer 1, we did not think it was feasible to include this all in a single manuscript. And in line with the descriptive nature of this study’s objective, and to minimise the risk of misinterpretation, we selected age as the primary variable for the analysis here.**

Instead of the sex-based distribution presented in Table 1, considering the distribution by age and educational attainment could reveal significant inequalities. Furthermore, analyzing the interplay between educational attainment, employment status (employed/unemployed), and gender would facilitate a more comprehensive understanding of labor inclusion challenges within this setting. Isolated data, such as the percentage of female employment in the public sector, without stratification by educational level, lacks the necessary depth to inform context-sensitive intervention strategies for low-resource settings.

**We believe it is important to keep the sex distribution in Table 1. As the SAGER Guidelines state (doi: 10.1186/s41073-016-0007-6), “*data should be routinely presented disaggregated by sex and gender. Sex- and gender-based analyses should be reported regardless of positive or negative outcome*.” While we agree that future studies could benefit from a more detailed analysis of the interaction between educational attainment, and employment status, we maintain that presenting the data in Table 1 disaggregated by sex/gender remains relevant.**

**Evidence suggests that the sex/gender system always intersects with other social axes of inequality in shaping health outcomes. For this reason, our decision to present sex-disaggregated data both in table 1 description of the socioeconomic characteristics of the study population and the NCD risk factors, reflects the study’s scope and the importance of highlighting gendered patterns in NCD risk factors within this underserved urban context.**

This is even more so when their conclusions state the following: "The established links between hypercholesterolaemia, diabetes, and hypertension with unhealthy patterns and pathogenic environments suggest that actions should be defined to address the social determinants of health, incorporating insights from various fields to reduce inequalities, particularly gender inequalities, and create fair environments that promote and improve health of men and women".

**We have restructured the discussion section to better align our conclusions with the evidence presented in our results. We now highlight specific findings that justify the need for broad population actions that create fairer and more health-promoting environments.**

It would be valuable to consider age stratification with ranges allowing for enhanced granularity in the prevalence analysis, particularly within groups exhibiting dynamic consumption patterns (Table 2). For instance, disaggregating age cohorts into Young Adults (18-25 y), Early Adults (25-35 y), Middle-Aged Adults (35-60 y), and Older Adults (60 years and older) would be beneficial. Specifically, further disaggregation of the 18–40-year range into narrower subgroups, such as 18-25 years, could elucidate specific trends in young adulthood, a stage where, as noted, consumption is often influenced by autonomy, university/work life, and social determinants, including risky behaviors like binge drinking and social smoking.

**We found your comment highly relevant and fully agree on the importance of enhancing granularity in the analysis, particularly for behavioural risk factors where age in early life can reveal critical patterns. In response, we have included a more detailed analysis in the supplementary material and described prevalences according to the recommended age groups and included references in results. Due to the observed similarities in prevalence between some adjacent groups (e.g. young adults and early adults), we have opted to retain the original age groupings in the main presentation of the manuscript. These groups coincide with those defined in the other STEPS surveys, including the previous Ecuadorian one, which improves comparability of findings.**

- To enhance the interpretation of metabolic risk factor outcomes (Table 3), additional stratification by nutritional status, beyond age cohorts, would be valuable. As evidenced by prior research, including recent studies in the Ecuadorian population lipid profiles and other metabolic parameters can vary significantly according to nutritional status. Incorporating this stratification could facilitate the identification of specific subpopulations within age ranges exhibiting distinct metabolic risk profiles, thereby informing more targeted interventions.

Ref: Albuja Quintana, Natalia (2025) Relationship between plasma uric acid levels, antioxidant capacity, and oxidative damage markers in overweight and obese adults: A cross-sectional study. <https://doi.org/10.1371/journal.pone.0312217>

**We found your comment highly valuable. Given that nutritional status emerged as a key result in our analysis, we decided to include an additional table in the supplementary material where overweight and obesity are examined across different age groups. This allows for a more detailed interpretation of metabolic risk factors by age and nutritional status, and supports the identification of subpopulations with distinct risk profiles.**

In the discussion section, the authors excessively elaborate on mechanisms that their own data cannot substantiate, as exemplified in lines 295-305, 312-318, 328-336, and 341-345. Given the cross-sectional design and the limited scope of statistical analyses, mechanistic explanations are challenging to support without additional experimental evidence. It would be more appropriate for the authors to reorganize the discussion section based on their own findings to clearly articulate the novelties or actual conclusions of this study to the readers.

**We have restructured the discussion section to better reflect the cross-sectional nature of our findings. We have revised the text to ensure that the discussion remains grounded in our own results, focusing on the descriptive nature of the data and clearly outlining the main contributions and novel aspects of the study.**

**In this regard, the ubiquitous nature of these risk factors also points to the influence of broader structural and commercial determinants of health. While we acknowledge that our study was not designed to directly test causal mechanisms, we have retained references to structural and commercial determinants of health—albeit in a more cautious form—to reflect the broader context in which these risk factors emerge. We believe it is important to situate our findings within this wider context and to recognize the need for sustained political and multisectoral action to address the upstream conditions that shape NCD risk in marginalized populations. Furthermore, the gender differences observed, underscore the importance of adopting an integrated, equity-oriented lens when designing public health responses.**

- Starting on line 73, the bibliographic citation has a different format that should be corrected, from ref 12 to ref 17.

**Corrected**

- Line 341: The information is repeated

These findings highlight. These findings highlight the high burden o…

**Corrected**

- Figure 2: Does it not describe the units of measurement for each parameter, for example, total cholesterol is in mg/dl?

Corrected

**Supplementary comment about the models and the variability of interactions.**

**Dear reviewers, as mentioned earlier in our response, we acknowledge that exploring the influence of multiple sociodemographic variables, but we chose to maintain the descriptive approach according to age and sex in the main manuscript. More in-depth analyses are currently being conducted for individual risk factors. For example, when we look at obesity, we feel there is a need to apply an intersectional lens to understand how gender, education, income, employment and age interact and are currently preparing a manuscript with this focus.**

**As shown in Tables 1, 2, and 3, we conducted univariable logistic regression analyses using Stata to examine the associations between hypertension, hyperglycaemia, and hypercholesterolaemia and each sociodemographic variable included in the study. For each variable, we reported the odds ratio (OR), 95% confidence interval (CI), and corresponding p-value. Subsequently, all variables were entered into a multivariable logistic regression model (aOR) using a stepwise selection procedure in Stata, with a significance level of 0.05 set as the criterion for variable retention (-**stepwise, pr(0.05) pe(0.05):logistic-**). Age emerged as the principal variable associated with the outcomes in the adjusted model.**

Table 1. Univariate regression model and multivariate model by sociodemographic variables for hypertension condition

|  | **OR** | **95%CI** | **p** | **aOR** | **95%CI** | **p** |
| --- | --- | --- | --- | --- | --- | --- |
| **Sex** |  |  |  |  |  |  |
| Male | 1.00 | - |  | 1.00 | - |  |
| Female | 0.60 | 0.41-0.89 | **0.011** | 0.59 | 0.38-.91 | **0.019** |
| **Age** | 1.07 | 1.0-1.09 | **<0.001** | 1.07 | 1.05-1.09 | **<0.001** |
| **Education** |  |  |  |  |  |  |
| Primary school | 1.00 | - |  | - | - | - |
| Secondary school and technical | 0.51 | 0.34-0.78 | **0.002** | - | - | - |
| University studies | 0.56 | 0.33-0.97 | **0.039** | - | - | - |
| **Marital status** |  |  |  |  |  |  |
| Single | 1.00 | - |  | - | - | - |
| Married | 1.19 | 0.82-1.74 | 0.345 | - | - | - |
| **Employment** |  |  |  |  |  |  |
| Unemployed | 1.00 | - |  | - | - | - |
| Unable to work | 0.51 | 0.34-0.78 | **0.002** | - | - | - |
| Employed | 0.56 | 0.33-0.97 | **0.039** | - | - | - |
| **Monthly income** |  |  |  |  |  |  |
| [0-400) | 1.00 | - |  | - | - | - |
| [400-800) | 0.50 | 0.31-0.80 | **0.004** | - | - | - |
| ≥800 | 0.81 | 0.45-1.45 | 0.487 | - | - | - |

Table 2. Univariate regression model and multivariate model by sociodemographic variables for Hyperglycaemia condition

|  | **OR** | **95%CI** | **p** | **aOR** | **95%CI** | **p** |
| --- | --- | --- | --- | --- | --- | --- |
| **Sex** |  |  |  |  |  |  |
| Male | 1.00 | - |  | - | - | - |
| Female | 1.21 | 0.62-2.37 | 0.572 | - | - | - |
| **Age** | 1.05 | 1.03-1.07 | **<0.001** | 1.06 | 1.03-1.09 | **<0.001** |
| **Education** |  |  |  |  |  |  |
| Primary school | 1.00 | - |  | - | - | - |
| Secondary school and technical | 0.41 | 0.21-0.81 | **0.010** | - | - | - |
| University studies | 0.46 | 0.18-1.12 | **0.090** | - | - | - |
| **Marital status** |  |  |  |  |  |  |
| Single | 1.00 | - |  | - | - | - |
| Married | 0.79 | 0.43-.1.47 | 0.471 | - | - | - |
| **Employment** |  |  |  |  |  |  |
| Unable to work | 1.00 | - |  | - | - | - |
| Unemployed | 0.04 | 0.00-0.38 | **0.004** | - | - | - |
| Employed | 0.29 | 0.13-0.64 | **0.002** | - | - | - |
| **Monthly income** |  |  |  |  |  |  |
| [0-400) | 1.00 | - |  | - | - | - |
| [400-800) | 0.60 | 0.29-1.23 | 0.166 | - | - | - |
| ≥800 | 0.33 | 0.09-1.16 | 0.086 | - | - | - |

Table 3. Univariate regression model and multivariate model by sociodemographic variables for Hypercholesterolemia condition

|  | **OR** | **95%CI** | **p** | **OR** | **95%CI** | **p** |
| --- | --- | --- | --- | --- | --- | --- |
| **Sex** |  |  |  |  |  |  |
| Male | 1.00 | - |  | - | - | - |
| Female | 0.98 | - | 0.917 | - | - | - |
| **Age** | 1.03 | 1.02-1.47 | **<0.001** | 1.03 | 1.02-1.05 | **<0.001** |
| **Education** |  |  |  |  |  |  |
| Primary school | 1.00 | - |  | - | - | - |
| Secondary school and technical | 0.49 | 0.32-0.73 | **0.001** | - | - | - |
| University studies | 0.57 | 0.35-0.95 | **0.031** | - | - | - |
| **Marital status** |  |  |  |  |  |  |
| Single | 1.00 | - |  | - | - | - |
| Married | 0.79 |  | 0.357 | - | - | - |
| **Employment** |  |  |  |  |  |  |
| Unable to work | 1.00 | - |  | - | - | - |
| Unemployed | 0.33 | 0.15-0.71 | **0.005** | - | - | - |
| Employed | 0.78 | 0.41-1.47 | 0.448 | - | - | - |
| **Monthly income** |  |  |  |  |  |  |
| [0-400) | 1.00 | - |  | - | - | - |
| [400-800) | 0.86 | - | 0.478 | - | - | - |
| ≥800 | 0.70 | - | 0.203 | - | - | - |
