# Supplementary material for: The burden of non-communicable disease risk factors in a low-income population: Findings from a cross-sectional study highlighting the prevalence of obesity, hypertension, and metabolic disorders in the south of Quito, Ecuador
Source: PLoS One. 2025 Sep 17;20(9):e0332159. doi: 10.1371/journal.pone.0332159 (PMC12443244; doi:10.1371/journal.pone.0332159)
Supplement: Attachment — Submitted filename: responsetoreviewers.docx [file pone.0332159.s006.docx]

**Dear reviewers, we thank your time and patience on reviewing a second time our manuscript, we are sure that all your reccomendations and advice increase the quality of this research.**

Reviewer #1:

Line 33: Please include details on the sample size and power.

**We completely agree on the importance of this issue, so we decided to include a detailed description of our sample characteristics in lines 33–35, which states: “We used multi-stage cluster sampling to select 656 of población total adult residents of 17D06 health district, enabling a prevalence estimation with at least ±5.73% absolute precision.” and in lines 136-144 a broader description of the sample method use.**

Line 47. Please describe comparisons among the main results and other similar studies in Latin American countries.

**We included a phrase in the conclusion section where we compare our results, specifically in lines 48–49, which state: “The critical prevalence of NCD risk factors in this low-income urban district of Quito, alongside similar trends observed in other settings across Latin America, underscores...”**

Line 51: Add keywords describing the site: Ex, Ecuador, Latin American countries.

**Included**

Line 110: add indicators about environmental exposures and vulnerability.

**We decided to include some indicator of exposure, impact and poverty segregation characteristichs of Quito in lines 117-127.**

Line 127. Figure 1: include latitude and longitude; make edits for improving visibility.

**We decided to reestructure the referenced map includign suggestions.**

Line 131: Describe the selection criteria for the participants.

**Due to the nature of this population survey, all people over 18 resident in the 17D06 district were elegible for the inclusion. Inclusion criteria was age and residence. We included a phrase were we stated it in lines 135**

Line 147: Review the figure number. Add a food note with details of the 319 refusal participants. Add a table about their descriptive statistic and the implications of the obtained prevalences (biases).

**We acknowledge the importance and the need to describe why some individuals decided not to participate. Unfortunately, when we collected the information in our field notes, the survey team recorded cases of rejection but did not capture detailed reasons. Qualitatively, they reported a high proportion of refusals linked to fear of COVID-19, or individuals who simply did not open the door due to concerns about insecurity. For this reason, we are unable to provide a table as suggested. However, we recognise the limitation this represents, particularly regarding potential biases in the prevalence estimates. We have expanded our description of this limitation in the discussion section in lines 404–408.**

Line 166: Please consider including the questionnaire in the supplementary material.

**We decided to include the questionnarie in supplementary material refered in lines 186-187**

Line 264: Please clarify if the monetary unitary is dollars or another currency.

**The currency was dollars, we included the currency.**

Line 345: Add the units for each axis.

**Added**

General comments
It could be useful to include a list of cut-off points for each biochemical definition.
Use a point to indicate decimal values.

**Thank you for the observation. The table detailing the cut-off points for the variables is already included as supplementary material and refered in the main text in lines 239-240. Additionally, the commas have been as suggested.**
